# Chemical transformation of xenobiotics by the human gut microbiota

**DOI:** 10.1126/science.aag2770

**Published:** 2018-08-24

**Authors:** Nitzan Koppel, Vayu Maini Rekdal, Emily P. Balskus

**Affiliations:** 1 Department of Chemistry and Chemical Biology, Harvard University, 12 Oxford Street, Cambridge, MA 02138, USA; 2 Broad Institute, Cambridge, MA 02139, USA

## Abstract

The human gut microbiota makes key contributions to the metabolism of ingested compounds (xenobiotics), transforming hundreds of dietary components, industrial chemicals, and pharmaceuticals into metabolites with altered activities, toxicities, and lifetimes within the body. The chemistry of gut microbial xenobiotic metabolism is often distinct from that of host enzymes. Despite their important consequences for human biology, the gut microbes, genes, and enzymes involved in xenobiotic metabolism are poorly understood. Linking these microbial transformations to enzymes and elucidating their biological effects is undoubtedly challenging. However, recent studies demonstrate that integrating traditional and emerging technologies can enable progress toward this goal. Ultimately, a molecular understanding of gut microbial xenobiotic metabolism will guide personalized medicine and nutrition, inform toxicology risk assessment, and improve drug discovery and development.

**BACKGROUND:**

Humans ingest a multitude of smallmolecules that are foreign to the body (xenobiotics), including dietary components, environmental chemicals, and pharmaceuticals. The trillions of microorganisms that inhabit our gastrointestinal tract (the human gut microbiota) can directly alter the chemical structures of such compounds, thus modifying their lifetimes, bioavailabilities, and biological effects. Our knowledge of how gut microbial transformations of xenobiotics affect human health is in its infancy, which is surprising given the importance of the gut microbiota. We currently lack an understanding of the extent to which this metabolism varies between individuals, the mechanisms by which these microbial activities influence human biology, and how we might rationally manipulate these reactions. This deficiency stems largely from the difficulty of connecting this microbial chemistry to specific organisms, genes, and enzymes.

**ADVANCES:**

Over the past several decades, studies of gut microbiota–mediated modification of xenobiotics have revealed that these organisms collectively have a larger metabolic repertoire than human cells. The chemical differences between human andmicrobial transformations of ingested compounds arise not only from the increased diversity of enzymes present in this complex and variable community but also from the distinct selection pressures that have shaped these activities. For example, whereas host metabolism evolved to facilitate excretion of many xenobiotics from the body, microbial modifications of these compounds and their human metabolites often support microbial growth through provision of nutrients or production of energy. Notably, the chemistry of microbial transformations often opposes or reverses that of host metabolism, altering the pharmacokinetic and pharmacodynamic properties of xenobiotics and associated metabolites.

The range of xenobiotics subject to gutmicrobial metabolism is impressive and expanding. Gut microbes modify many classes of dietary compounds, including complex polysaccharides, lipids, proteins, and phytochemicals. These metabolic reactions are linked to a variety of health benefits, aswell as disease susceptibilities. Gut microbes are also able to transform industrial chemicals and pollutants, altering their toxicities and lifetimes in the body. Similarly, microbial transformations of drugs can change their pharmacokinetic properties, be critical for prodrug activation, and lead to undesirable side effects or loss of efficacy. In the vast majority of cases, the individual microbes and enzymes that mediate these reactions are unknown.

Fueled by findings underscoring the relevance of microbial xenobiotic metabolism to human health, scientists are increasingly seeking to discover and manipulate the enzymatic chemistry involved in these transformations. Recent work exploring how gut microbes metabolize the drugs digoxin and irinotecan, as well as the dietary nutrient choline, provides guidance for such investigations. These studies, which combine traditional methods with modern approaches, illustrate how a molecular understanding of gut microbial xenobiotic metabolism can guide hypothesis-driven research into the roles these reactions play in both microbiota and host biology.

**OUTLOOK:**

We still face a myriad of challenges in understanding the gut microbiota’s contribution to xenobiotic metabolism. It is imperative that we connect the many known microbial transformations with the genes and enzymes responsible for these activities, and knowledge of enzyme mechanism and biochemical logic will facilitate this objective. There also remains a great need to uncover currently unappreciated activities associated with this community. Revealing the full scope of microbially mediated transformations in the gut may give us newinsights into themany variable and contradictory studies regarding the effects of diet, pollutants, and drugs on human health. Microbial genes and enzymes will provide both specific targets for manipulation and diagnostic markers that can be incorporated into clinical studies and practice. Ultimately, a molecular understanding of gut microbial xenobiotic metabolism will inform personalized nutrition, toxicology risk assessment, precision medicine, and drug development.

**Figure:**
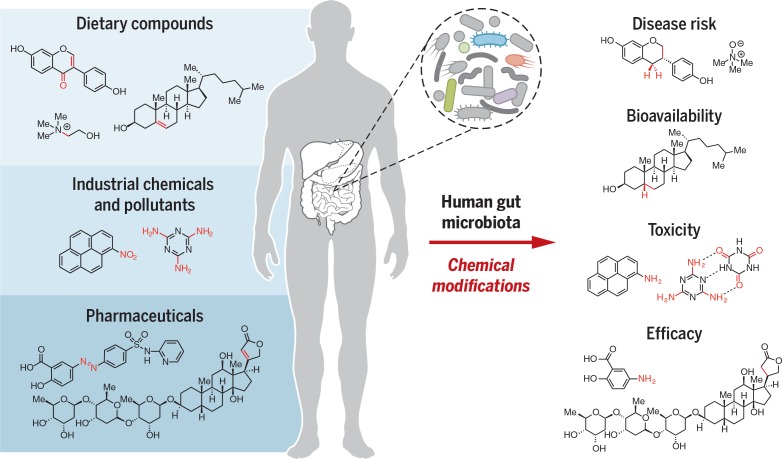
**Human gut microbes metabolize xenobiotics.** Themicroorganisms that inhabit the human gut alter the chemical structures of ingested compounds, including dietarycomponents, industrial chemicals, and drugs.These changes affect xenobiotic toxicity, biological activity, and bioavailability.The gutmicrobialenzymesresponsibleformanyofthesetransformationsarepoorlyunderstood.Me,methyl.

The human gut microbiota is a diverse and complex community of microorganisms that has evolved with its host (*1*) and is deeply intertwined with human biology. The estimated 1013 microbes that inhabit the human gastrointestinal (GI) tract play a central role in many processes, including colonization resistance, immune system modulation, synthesis of essential vitamins and nutrients, and digestion of polysaccharides (*2*–*4*). Gut microbes also modify the chemical structures of numerous ingested, foreign compounds (xenobiotics), including dietary components, environmental pollutants, and pharmaceuticals. Such transformations were identified as early as the 1950s in humans, animal models, fecal samples, and individual microbes. In these studies, changes in metabolism in the absence of microbes [i.e., germ-free (GF) animals] or upon microbial perturbation (i.e., antibiotic treatment or dietary modulation) indicated the gut microbiota’s involvement in xenobiotic processing (*5*, *6*).

The human gut microbiota encodes a broad diversity of enzymes, many of which are exclusivelymicrobial, thus expanding the repertoire of metabolic reactions occurring within the human body. Although clinical studies have revealed marked interindividual variability in these microbial transformations, most of these reactions have consequences for the host that are not completely understood. Gut microbial xenobioticmetabolites are known to have altered bioactivity, bioavailability and toxicity and can interfere with the activities of human xenobiotic-metabolizing enzymes to affect the fates of other ingested molecules. Despite the diverse and physiologically important consequences of these modifications, relatively little is known about the specific gut microbial strains, genes, and enzymes that mediate xenobiotic metabolism.

Here we review our current knowledge of how the gut microbiota directly modifies dietary compounds, environmental pollutants, and pharmaceuticals. We discuss critical differences between the chemistry of microbial and host xenobiotic metabolism and highlight opportunities for enzyme discovery and characterization. By examining several recent studies, we show how gaining a molecular understanding of microbial xenobiotic modifications can illuminate their connections to human health. Finally, we discuss strategies for uncovering the genetic and biochemical bases of these microbial activities and provide an outlook on how understanding gut microbial xenobiotic metabolism will influence personalized nutrition, toxicology, and medicine.

## Gut microbial interactions with xenobiotics

The majority of human microbiota-xenobiotic interactions occur within the GI tract. The different regions of this organ system vary in epithelial cell physiology, pH, oxygen levels, and nutrient content, thus providing distinct habitats for microorganisms and influencing the types of metabolic processes that occur (*7*, *8*). Hundreds of distinct microbial species colonize the human gut. Although obligate anaerobes such as the Firmicutes and Bacteroidetes phyla typically dominate, large variability in community composition is observed among individuals (*9*, *10*).

Microbial metabolism of xenobiotics must be understood in the context of the concurrent and often competing metabolic processes occurring in the human host. Orally ingested compounds pass through the upper GI tract to the small intestine where they can be modified by digestive enzymes and absorbed by host tissues (*11*). Readily absorbed xenobiotics pass between or through intestinal epithelial cells, where they may be processed by host enzymes before transport to the liver via the portal vein. Following exposure to the liver’s rich collection of metabolic enzymes, xenobiotics and their metabolites enter systemic circulation, distributing into tissues and potentially affecting distal organs. By contrast, intravenously administered compounds circumvent this “firstpass” metabolism and are immediately introduced into systemic circulation. Compounds in the circulatory system are eventually further metabolized and/or excreted, which generally occurs either via the biliary duct back into the gut lumen (biliary excretion) or through the kidneys into the urine. Metabolites returned to the gut lumen can either continue on to the large intestine, where they will eventually be excreted in the feces, or they can potentially be reabsorbed by host cells in the small intestine through a process known as enterohepatic circulation.

Xenobiotics can therefore encountergutmicrobes via multiple routes. In contrast to compounds that are absorbed in the small intestine, poorly absorbed xenobiotics continue through the small intestine into the large intestine and may be transformed by gut microbes. Readily absorbed compounds and compounds administered via other routes (e.g., intravenous injection) can also reach gut microbes through biliary excretion. The products of gut microbialmetabolism can be absorbed by the host and circulated systemically or interact locally with the epithelial cells lining the GI tract. Ultimately, these microbial metabolites are excreted in feces or filtered by the kidneys and eliminated in the urine. Overall, human and microbial transformations generate a complex intertwined metabolic network that affects both the host and the members of the microbiota.

## The complementary chemistry of microbial xenobiotic metabolism

Within the distinctive and complex ecology of the human gut, microorganisms transform ingested substrates via a broad range of enzymatic reactions. Gut microbes primarily use hydrolytic and reductive reactions tometabolize xenobiotics (*5*), many of which are distinct to these organisms. This is in stark contrast to host enzymes, which typically use oxidative and conjugative chemistry. These differences are partially due to physiological context, but they also reflect distinct evolutionary pressures. Additionally, it is likely that certain gut microbial enzymes were not evolutionarily selected to process specific xenobiotics, but rather that metabolism arises from relaxed substrate specificity. Thus, the combined metabolisms of host and microbiota generate metabolites that would not be synthesized by the host alone and can substantially alter the bioactivities and lifetimes of xenobiotics within the human body.

Many of the enzyme classes associated with xenobiotic metabolism (hydrolases, lyases, oxidoreductases, and transferases) and highlighted here are widely distributed among sequenced gut microorganisms (*12*–*16*). Metagenomic analyses have also revealed them to be among the most prevalent protein families in this environment (*17*, *18*). It is therefore likely thatmany important transformations of xenobiotics may be performed by multiple different phylogenetic groups of gut microbes.However, it is critical to note that broad annotations are not predictive of substrate specificity, as enzymes with high sequence similarity can catalyze distinct chemical reactions.Metabolic activities can also be discontinuously distributed across closely related strains and acquired via horizontal gene transfer, making it problematic to infer gut microbial metabolic capabilities from phylogenetic analyses alone. This issue highlights the value of culture-based experiments and rigorous biochemical characterization of gut microbial enzymes in understanding xenobiotic metabolism.

### Host xenobiotic metabolism

Any discussion of microbial xenobiotic transformations must also consider host chemical capabilities. Human xenobioticmetabolism generally transforms nonpolar compounds into hydrophilic, higher–molecular weightmetabolites that aremore readily excreted (Box 1A). This process occurs in two stages: installation or exposure of polar functional groups (“phase I”) and conjugation of these groups to more-polar metabolites (“phase II”). Phase I enzymes perform oxidative, reductive, or hydrolytic reactions to generate hydroxyl groups, epoxides, thiols, and amines. The largest class of phase I enzymes is the cytochrome P-450s, but carboxylesterases and flavin monooxygenases (FMOs) are also important in xenobiotic processing (*11*). Transferase enzymes predominate in phase IImetabolism, appending glucuronyl,methyl, acetyl, sulfonyl, and glutathionyl groups onto xenobiotics or phase Imetabolites (*19*). Polymorphisms in xenobiotic-metabolizing genes influence how individuals respond to both dietary and pharmaceutical interventions.

Box 1 **The chemistry of gut microbial and host xenobiotic metabolism.** (**A**) Chemical logic of host xenobiotic metabolism. Commonly used chemical strategies for microbial xenobiotic metabolism include (**B**) hydrolytic transformations, (**C**) lyase reactions, (**D**) reductive transformations, (**E**) functional group transfer reactions, and (**F**) transformations mediated by radical enzymes. Enz, enzyme; PLP, pyridoxal 5-phosphate; NAD(P)H, NADH or NADPH; FAD, flavin adenine dinucleotide; FMN, flavin mononucleotide; Me, methyl; CoA, coenzyme A; SAM, *S*-adenosylmethionine.

**Figure:**
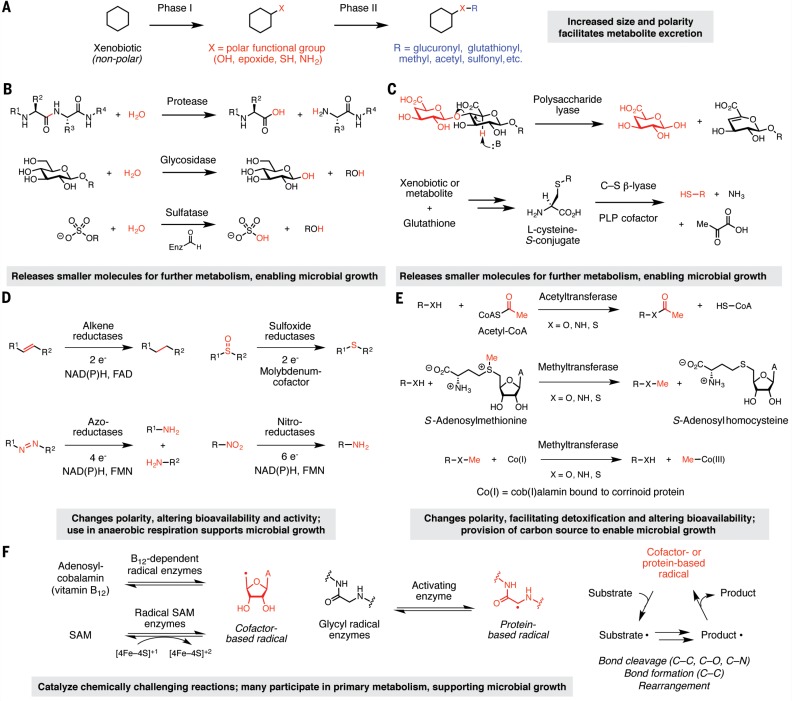

### Hydrolytic reactions

Both the host and gut microbiota use hydrolytic chemistry to break down large ingested compounds into smaller products that may be further metabolized. Hydrolase enzymes catalyze the addition of a water molecule to a substrate, followed by bond cleavage (Box 1B). The most abundant and relevant hydrolases in the GI tract are proteases, glycosidases, and sulfatases, with the microbiota contributing a broader range of activities than host enzymes. Proteases cleave the peptide bonds linking the amino acids in polypeptide chains. Whereas the small intestine is dominated by pancreatic serine proteases, the colon contains many microbial cysteine- and metalloproteases (*20*) with different substrate specificities and potentially different clinical consequences (*21*). Glycosidases hydrolyze glycosidic bonds using a dyad of carboxylic acid residues and a water molecule, releasing free sugars (*22*). These enzymes process a huge diversity of glycoconjugates and oligosaccharides and are broadly distributed across gut microbes (*15*, *23*). Sulfatases, which are also widespread, hydrolyze sulfate esters generated by phase II host metabolism using the unusual amino acid formylglycine (*24*). The hydrate form of this residue is thought to undergo transesterification with a sulfate ester substrate to generate a tetrahedral intermediate that breaks down to release sulfate and reform the aldehyde (*25*).

Hydrolytic reactions alter both the physical properties and activities of xenobiotics and their metabolites. For example, removal of a glucuronide in the gut lumen is generally accompanied by a decrease in polarity that can allow reabsorption by host cells and thereby extend the lifetime of a molecule within the body, as seen with glucuronide conjugates of nonsteroidal antiinflammatory drugs and the cancer therapy irinotecan (*26*, *27*). Hydrolysis can also alter the biological activity or toxicity of xenobiotics, as observed for plant-derived glycosides like amygdalin and the artificial sweetener cyclamate (*28*, *29*). Moreover, hydrolysis is often a prerequisite for further transformations, such as the fermentation of sugars released from indiges indigestible polysaccharides (*30*), and the products of hydrolytic reactions (sugars, amino acids, and sulfate) often support microbial growth and survival in the gut.

### Lyases

Lyase enzymes break C–C or C–X bonds (where X = O, N, S, P, or halides) without relying on oxidation or the addition of water. Microbial polysaccharide lyases (PLs) modify polysaccharides that contain a glycosidic bond at the β position relative to a carboxylic acid (e.g., alginate, pectin, chondroitin, and heparan) (Box 1C). The presence of the carboxylate enables removal of the a proton and subsequent β-elimination to yield an α,β-unsaturated sugar and a hemiacetal (*12*). El Kaoutari *et al*. found that a single human gut microbiome encoded >5000 PLs (*15*), suggesting an enormous diversity of transformations that could support microbial growth.

Microbial C–S β-lyases cleave C–S bonds found in both dietary compounds and cysteine-*S*-conjugates of xenobiotics, which are formed by liver enzymes (Box 1C). These enzymes generate an aldimine linkage between pyridoxal 5-phosphate (PLP) and the a-amino group of the cysteine-derived substituent, acidifying the adjacent proton. β-elimination releases a thiol-containing metabolite and aminoacrylate, the latter of which spontaneously breaks downto formammonia and pyruvate (*31*). Microbes can further metabolize these thiols, altering their physical properties and localization within the body. For example, gut bacterial C–S β-lyases cleave cysteine-*S*-conjugates of polychlorinated biphenyls to produce thiol metabolites that are further methylated and accumulate in lipophilic host tissues (*32*). The consequences of C–S lyase chemistry for the microbiota are not well understood. This activitymay derive from promiscuous PLP-dependent enzymes involved in “housekeeping” functions (*31*); however, ammonia generated by C–S β-lyases can serve as a sole nitrogen source (*33*), pointing to a potential role in nutrient acquisition.

### Reductive transformations

Gut microbes can reduce a wide range of functional groups, including alkenes and α,β-unsaturated carboxylic acid derivatives, nitro, *N*-oxide, azo, and sulfoxide groups (Box 1D). Reductase enzymes make use of various cofactors [e.g., NAD(P)H (i.e., NADH or NADPH), flavin, Fe–S clusters, (siro) heme, molybdenum cofactor, and other metallocofactors] to mediate the transfer of electrons or hydride equivalents (H^+^, 2e^–^) to substrates (*34*–*36*). Biochemical and structural characterization of gut microbial reductases has uncovered individual enzymes that display broad substrate scope and can transform multiple functional groups; consequently, their endogenous substrates and in vivo relevance are often unclear.

Reduction typically decreases the polarity of compounds and can alter charge, hybridization, and electrophilicity, which can affect the lifetimes and activities of metabolites in the body (*37*–*39*). Notably, electron transfer to xenobiotics may enable anaerobic respiration in the human gut, where oxygen is largely unavailable to serve as a terminal electron acceptor. Although reductase enzymes are found in humans, many reductive transformations are exclusively microbial (*6*, *37*, *40*) and have not yet been linked to known enzymes or organisms.

### Functional group transfer reactions

Transferase enzymes move functional groups between two substrates via nucleophilic substitution reactions. The gut microbiota transfers methyl and acyl groups to or from xenobiotic scaffolds (Box 1E). Addition reactions require chemically activated cosubstrates, such as acetyl coenzyme A, adenosine triphosphate (ATP), or *S*-adenosylmethionine (SAM).Whereas enzymes that remove acyl groups generally rely on hydrolysis, demethylating enzymes use cofactors capable of nucleophilic catalysis [e.g., cob(I)alamin and tetrahydrofolate] (*41*, *42*). Installation and removal of these functional groups can affect the lifetimes and bioactivities of xenobiotics in various ways. For instance, acetylation can serve as a detoxification mechanism by decreasing polarity and facilitating excretion from microbial cells. A notable example is the N-acetylation of the anti-inflammatory compound 5-aminosalicylic acid (5-ASA) by microbial *N*-acetyltransferases (*43*), which yields a therapeutically inactive metabolite. Host demethylation of xenobiotics liberates polar groups for further conjugation and excretion from the body (*44*), but in microorganisms, demethylation can provide a carbon source for growth (*41*).

### Radical chemistry

Radical enzymes generate high-energy intermediates containing an unpaired electron. Such processes are often oxygen-sensitive and energetically demanding, but enable microbes to perform chemically challenging reactions that are inaccessible by other modes of catalysis, including bond cleavage and formation (both C–C and C–X, where X = N, O, or halides) and skeletal rearrangements (*45*). Many radical enzymes used in anaerobic metabolism share a common chemical logic. By using an enzyme- or cofactor-based radical species, these enzymes typically generate a substrate-based radical intermediate through single-electron transfer or homolytic bond cleavage (Box 1F). This initial substrate-based radical is then converted to a product-based radical. Formation of the final product often regenerates the initial enzyme- or cofactor-based radical to complete the catalytic cycle.

Key classes of gut microbial radical enzymes include radical SAM enzymes, cobalamin (B_12_)– dependent enzymes, and glycyl radical enzymes (GREs). These enzymes often mediate primary metabolism in anaerobic microbes and can directly or indirectly influence the fate of xenobiotics in the human body. Reactions catalyzed by gut microbial GREs include generation of trimethylamine (TMA) by choline trimethylaminelyase (CutC) (*46*) and decarboxylation of the tyrosine-derived metabolite *p*-hydroxyphenylacetate by *p*-hydroxyphenylacetate decarboxylase (*47*). This latter reaction produces *p*-cresol, amolecule that competes with xenobiotics for O*-*sulfation and detoxification by host enzymes (*48*).

### Uncharacterized xenobiotic metabolism

The vast majority of gut microbial xenobiotic transformations cannot be linked to specific enzymes and organisms.Whereas certain reactions can, with reasonable confidence, be associated with one of the enzyme classes highlighted above, other uncharacterized metabolic activities cannot be readily explained with known biochemistry. Below we discuss prominent gaps in our knowledge of gut microbial xenobiotic metabolism, focusing on transformations with intriguing but poorly understood links to human health. Although not comprehensive, this overview highlights particularly notable opportunities for gut microbial enzyme discovery and characterization.

## Metabolism of dietary compounds

Gut microbes process an enormous variety of dietary compounds to extract nutrients and energy (*49*). The types and extent of these modifications vary substantially among individuals, presumably due to differences in the presence and abundance of gut microbial enzymes, and the bioactivities of the resulting metabolites range from beneficial to acutely toxic. As much attention has been focused on microbial metabolism of complex, plant-derived polysaccharides (*4*, *50*, *51*), we have chosen to highlight transformations of noncarbohydrate dietary components.

### Dietary protein

Dietary protein is necessary for supplying humans with essential amino acids, but the source and amount of protein can vary substantially between different diets. The gut lumen is rich in both host and microbial proteases, and studies increasingly indicate that differential microbial proteolytic activity may directly contribute to human disease. For example, the gut microbiota is associated with celiac disease (CD), a common autoimmune disorder characterized by an inflammatory response to dietary gluten found in wheat-based foods. This proline-rich protein evades complete digestion by host proteases, resulting in the generation of high–molecularweight, immunogenic peptides. The gut microbiota may affect CD by altering gluten proteolysis. Fecal suspensions from healthy individuals and CD patients process gluten proteins and immunogenic peptides differently (*52*). For instance, gluten-derived peptides generated by *Pseudomonas aeruginosa*, an opportunistic pathogen in CD patients, are prone to translocation across themouse intestine and elicit an enhanced gluten-specific immune response in comparison with peptides produced by *Lactobacillus* spp. from healthy individuals (*53*). Identification of specific proteases responsible for gut microbial gluten processing could not only enable a better understanding of CD but also inform therapeutic interventions for this disease1, including enzymatic or probiotic treatments.

Gut microbes can also metabolize amino acids obtained from dietary protein, including Lphenylalanine, L-tyrosine, and L-tryptophan, into a range of bioactive products (*54*). For example, gut bacteria can metabolize L-tryptophan into many products, including the antioxidant indole- 3-propionic acid, the neurotransmitter tryptamine, and indole, the latter of which can undergo hydroxylation and sulfation by hepatic enzymes to generate the uremic toxin indoxyl sulfate (*55*–*57*).

### Dietary lipids

Variable gut microbial metabolism of lipids and lipid-derived compounds is associated with a variety of human diseases (*58*, *59*). One notable example involves dietary cholesterol, a major component of Western diets that is associated with increased risk of cardiovascular disease (*60*). While ingested cholesterol is absorbed in the small intestine and subsequently undergoes biliary excretion and enterohepatic circulation, gut microbial reduction of cholesterol generates coprostanol, which cannot be reabsorbed and is excreted. This transformation therefore effectively removes cholesterol from circulation. Coprostanol comprises up to 50% of the steroids in human feces (*61*), and GF mice colonized with microbes from high- and low-cholesterol–reducing patients produce distinct amounts of coprostanol (*62*). Animal experiments also suggest that cholesterolreducing bacteria may decrease serum cholesterol (*61*). Studies of the cholesterol-reducing gut bacteria *Eubacterium coprostanoligenes* indicate that coprostanol synthesis may involve oxidation to 5- cholesten-3-one followed by alkene isomerization to 4-cholesten-3-one, conjugate reduction, and ketone reduction (*63*). Identifying the enzymes responsible for these transformations and characterizing their abundance in patients may be particularly interesting, given that inhibition of cholesterol reabsorption is a clinically validated strategy for lowering cholesterol (*64*).

### Dietary phytochemicals

Identifying and characterizing gut microbial enzymes may also help us to better understand dietary compounds that are associated with health benefits. For example, numerous studies implicate the gut microbiota in metabolizing poorly absorbed, polyphenolic compounds from plantderived foods (*65*) including soy isoflavones, lignans from flaxseed and sesame seeds, flavonoids like the catechins and gallate esters found in tea, and ellagic acid from nuts and berries (*66*–*69*). These molecules are processed using a range of transformations, including ring cleavage, demethylation, and dehydroxylation, which generally produce metabolites with higher oral bioavailability, increased bioactivity, and a correlation with lowered disease risk (*67*, *68*). Polyphenolmetabolism varies widelyamong individuals (*67*), and further research is needed to elucidate whether these microbial products can directly affect host biology or are biomarkers for disease susceptibility. From a chemical standpoint, studying polyphenolmetabolismwill also address fundamental gaps in our understanding of gut microbial enzymes.

### Artificial sweeteners

The microbiome may also interact with components of our diets that are added in the process of food manufacturing (e.g., artificial sweeteners, emulsifiers, and preservatives). For instance, although many artificial sweeteners are poorly metabolized by humans, studies demonstrate that they are susceptible to microbial transformation. Gut microbes convert the artificial sweetener cyclamate into cyclohexylamine via hydrolytic cleavage of its sulfamate linkage. Cyclamate was banned in the United States after studies suggested that cyclohexylamine was carcinogenic, and both this finding and the continued use of this sweetener remain controversial (*70*). A cyclamate hydrolyzing enzyme has been partially purified from a guinea pig–associated strain, but human gut microbial hydrolases with this activity have not been identified (*29*). Gut microbes can also metabolize the artificial sweeteners stevioside and xylitol using unknown enzymes (*71*, *72*). The gut microbiota acquires the ability to transform xylitol and cyclohexamate after prolonged exposure, suggesting that long-term ingestion of dietary components can select for particular microbial metabolic functions (*73*). Understanding the microbiota’s role in metabolizing components of processed foods could be important for assessing food safety and the long-term effects of food additives on human health.

### Heterocyclic amines

Finally, an understanding of gutmicrobial metabolic activities could provide new insights into the biological consequences of cooking practices and food preparation. The mutagenic potential of heterocyclic amines, poorly absorbed molecules produced during charring of meat and fish, can be altered by gut microbial metabolism. For example, gut microbes convert 2-amino- 3-methylimidazo[4,5-*f*]quinoline (IQ) into the potentialmutagen 7-hydroxy IQ (*74*) and can hydrolyze IQ-glucuronide conjugates, prolonging the lifetime of IQ in the body (*75*). Gnotobiotic rats monoassociated with an *Escherichia coli* strain encoding a β-glucuronidase (*uidA*) have higher levels of unconjugated IQ and increased colonic DNA damage compared with rats colonized with an isogenic *uidA* mutant (*75*). These results appear to implicate the gut microbiota in the known link between charred meat and cancer (*76*).

## Metabolism of industrial chemicals and pollutants

Although there is an emerging appreciation for the role of the gut microbiota in metabolizing pollutants and industrial chemicals, our knowledge of the specific transformations, strains, and enzymes involved lags far behind that of environmental microbes. However, it is clear that microbial activities can alter the toxicity and bioavailability of these compounds, as well as extend host exposure to harmful substances. When evaluating the safety of these compounds, it is thus crucial to consider the consequences of gut microbial metabolism. Here we discuss several types of chemicals that have been implicated in human disease risk and for which there is evidence that gut microbial metabolism affects toxicity.

### Chemicals used in industrial manufacturing

Gut microbes reductively metabolize azo compounds, some of the first industrially important synthetic chemicals (*40*). Despite their use for >150 years as textile dyes, food colorings, and pharmaceuticals, we have an incomplete understanding of the organisms and enzymes that process these molecules. The reductive cleavage of azo linkages yields aniline products, and this reaction can be performed by flavin- or NAD(P)Hdependent enzymes found in many eukaryotes and bacteria (*13*). Azoreductases have not yet been extensively characterized from many human gut bacterial strains in which this activity has been observed, and isolates can vary in their ability to reduce different dyes (*77*). The biological consequences of azo reduction vary depending on the substrate. For example, although microbial transformation of azo food dyes generates metabolites that are considered to be nontoxic (*34*, *78*), workers with long-term exposure to textile dyes have an increased risk of bladder cancers (*79*). Feeding azo textile dyes to conventional, but not GF, mice leads to the accumulation of the mutagenic bis-aniline benzidine in urine, which implicates microbial metabolism in increasing carcinogen exposure (*80*). Thus, the toxic effects of azo compounds may depend on both the individual dye backbone and the presence of specific metabolizing organisms.

Gut microbes also metabolize the *s*-triazine compoundmelamine, an industrial chemical used in the production of various plastics, enhancing its toxicity in humans. Melamine added to infant formula in China caused kidney stones in 300,000 children and led to at least six deaths (*81*). Subsequent studies in mice revealed that gutmicrobes deaminate melamine to generate ammonia and cyanuric acid (*82*), the latter of which forms an insoluble complex with melamine in vivo, leading to renal toxicity (*83*). *Klebsiella* species are associated with cyanuric acid production in mice and generate this metabolite in vitro (*82*), but it remains unclear whether the gut microbiota or this organism contribute to melamine toxicity in humans. As environmental bacteria use similar hydrolytic chemistry to metabolize other industrially relevant *s*-triazines, including the herbicide atrazine, these studies also raise the possibility that the gut microbiota may transform additional compounds in this class.

### Heavy metals

In addition to organic pollutants, human gut microbes modify the structures and alter the toxicities of various heavy metals, including bismuth, arsenic, and mercury. Mercury bioaccumulates in living organisms, posing a threat to human health, and gut microbial metabolism may affect mercury toxicity and lifetime in the body. Rat fecal samples reduce methylmercury (CH3Hg+) to the less toxic inorganic mercury, thereby facilitating mercury excretion from the host (*84*). Depletion of the gut flora in rats and mice can result in the accumulation of methylmercury, thereby causing neurological symptoms (*85*). The enzymes responsible for this protective activity could include homologs of the demethylating, organomercuric lyase (MerB) and mercuric reductase (MerA), which have been identified in human isolates (*86*). However, the abundance of *mer* genes did not correlate with fecal methylmercury levels in a recent clinical study, raising the possibility of additional enzymes or indirect effects (*87*). Notably, incubation of a mixture of 16 metal(oid)s with suspensions from an in vitro simulator of the GI tract resulted in volatilization of manymetal species and the production of As/S compounds not previously observed in biological systems (*88*). These studies indicate that there aremajor gaps in our knowledge of the gut microbiota’s interactions with heavy metals and the resulting toxicological implications.

### Metabolism of pharmaceuticals

Apart from antibiotics, the human gut microbiota is known to transform >50 pharmaceuticals, spanning many indications and host targets, into metabolites with altered pharmacological properties (*5*, *89*). In some cases, the teratogenic (*90*), toxic (*26*, *27*), and even lethal (*91*) effects of these microbial metabolic activities were not recognized until drugs were on the market. Ongoing studies have illuminated a complex interplay between drugs and gut microbes (*92*, *93*), but we focus here on examples of direct microbial modifications.

### Anti-inflammatory and GI agents

Multipledrugs that target theGI tract are affected by gut microbes, either by direct chemical modification or indirectly through the many interactions these organisms have with host cells in this environment. Notably, many of these agents rely on microbial metabolism for converting inactive precursors (prodrugs) to pharmaceutically active compounds. Prominent examples are antiinflammatory drugs that contain azo linkages, including the inflammatory bowel disease (IBD) medication sulfasalazine (*38*). Gutmicrobes reduce sulfasalazine into sulfapyridine and the active antiinflammatory agent 5-ASA, and various intestinal bacteria can further metabolize 5-ASA into *N*-acetyl 5-ASA, a metabolite that lacks anti-inflammatory activity. Considerable variability in acetylation rates has been observed in human fecal samples (*94*). Together with differences in azo reduction, this observation could potentially explain variable therapeutic efficacy of sulfasalazine in patients. *N*-acetyl ASA inhibits the growth of anaerobes, including *Clostridium difficile* (*43*), which suggests that this activity could affect gut microbiome composition. This observation is particularly noteworthy given our increasing appreciation of the participation of the gut microbiota in IBD pathogenesis. Other gut microbial activities that are responsible for prodrug activation include reduction of the sulfoxide found in the anti-inflammatory compound sulindac (*95*) and reduction of the *N*-oxide of the anti-diarrheal drug loperamide (*39*). Gaining a better understanding of the specific organisms and enzymes responsible for these activities and their presence in patients could aid in drug selection and dosing.

### Cancer chemotherapy

Patient response to chemotherapy can differ dramatically between individuals, in terms of efficacy as well as the severity of side effects, and emerging studies suggest that differences in the gut microbiota may contribute to this phenomenon. In addition to modulating the host immune system, gut microbes can directly alter the structures of cancer therapies and their metabolites, affecting their interactions with host cells. Recent work indicates that microbes may have a high potential formodifying the chemical structures of commonly used chemotherapeutics (*96*). Co-incubation with either *E. coli* or *Listeria welshimeri* either increased or decreased the efficacy of half of a panel of 30 anticancer drugs toward cancer cell lines. Assays with *E. coli* and a subset of these drugs (gemcitabine, fludarabine, and CB1954) revealed evidence for direct chemical modification by the bacteria. Finally, the presence of *E. coli* altered the efficacy of chemotherapy in vivo in a manner consistent with the observed metabolic activities. These preliminary observations suggest that structural modification of drugs by gut or tumor-associated microbes could contribute to interindividual variation in cancer therapy, but examination of larger panels of microbes and detailed characterization of drug metabolites are needed to extend these findings.

### Central nervous system (CNS) drugs

In addition to affecting drugs that act locally, gut microbial metabolism can also influence the efficacy of therapeutics that target distant organ systems. Many prominent examples can be found among CNS drugs. For example, oral levodopa (L-dopa) is used to treat Parkinson’s disease, a condition characterized by dopaminergic-neuronal death. L-dopa crosses the blood-brain barrier,where it is decarboxylated by host enzymes to restore dopamine levels (*97*). However, extensive metabolism within the gut by both host and microbial enzymes affects the concentration of drug reaching the brain. Microbial decarboxylation (*98*) and *p*-dehydroxylation convert L-dopa to *m*tyramine, which can be further oxidized to *m*hydroxyphenylacetic acid (*99*). Differences in these activities may contribute to the substantial variation observed in patient response to L-dopa (*100*). Although a tyrosine decarboxylase from a foodassociated strain of *Lactobacillus brevis* accepts L-dopa in vitro (*101*), the human gut microbes and enzymes responsible for L-dopa metabolism are unknown. As studies continue to reveal connections between the gut microbiota and various neurological diseases (*102*), it will become increasingly important to identify and characterize additional microbial interactions with CNStargeted drugs.

## Herbal supplements and traditional medicines

Similar to dietary phytochemicals, gut microbes can also transform poorly absorbed constituents of herbal and traditional remedies, which can lead to potential health benefits or harmful side effects. There are many examples of the variable efficacy of traditional medicines, which may be partly due to the complexity of active ingredients, as well as to differences in gut microbial metabolism of these treatments. For example, amygdalin is amandelonitrile glycoside found in almonds and fruit pits that was used as an alternative cancer treatment in the 1960s (*103*), although clinical trials demonstrated no improvements in cancer survival or symptoms (*104*). In fact, subsequent animal experiments showed that gut microbes hydrolyze the glycosidic linkage of amygdalin to release mandelonitrile, which spontaneously breaks down to produce benzaldehyde and toxic cyanide (*28*, *105*). Gut microbes also metabolize the plant-derived benzoisoquinoline alkaloid berberine in a way that allows intestinal absorption (*106*) and convert ginsenosides, themajor bioactive components of ginseng, into metabolites that inhibit cytochrome P-450s and affect host xenobiotic metabolism (*107*). Because these remedies are not regulated to the same extent as pharmaceuticals, their modes of action are typically less well characterized. Elucidating how gut microbes process these compounds may contribute extensively to our fundamental understanding of their effects.

### Gut microbial biotransformation comes of age: Recent case studies

Attempts to decipher the biological consequences of gutmicrobial xenobiotic metabolism have been hindered by our poor understanding of these transformations. Although many associations exist between ingestion of xenobiotics processed by gut microbes and health status, we have limited information about the distribution of these activities in patient populations.Metabolic functions rarely correlate directly withmicrobial phylogeny, and considerable strain-level variation exists even within the same species (*108*), which limits the information gained from assessing the composition of the gut microbiota alone. Large-scale metagenomic analyses that claim to detect differences in putative xenobioticmetabolism pathways (*109*, *110*) have not provided experimental evidence supporting predicted changes in activities. To elucidate how gut microbial metabolism influences human health, it is critical to connect functions of interest with genes and enzymes. Recent studies show how the combination of traditional methods with emerging technologies can enable functional analysis. These examples also show how a molecular understanding of gut microbial xenobiotic metabolism can aid in manipulating these activities to benefit health outcomes.

### Digoxin: Identifying a predictive biomarker for drug metabolism

**Fig. 1:**
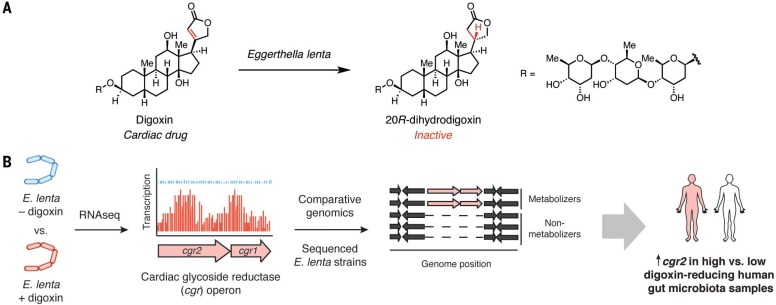
**Identifying gut microbial genes that predict cardiac drug metabolism. (A)** *E. lenta* reductive metabolism leads to cardiac drug inactivation. **(B)** A combination of culture-based studies, sequencing, and bioinformatics helped to identify microbial genes associated with digoxin metabolism in humans.

*Digitalis purpurea* (foxglove) plant extracts were first used to treat “dropsy” (congestive heart failure) more than 230 years ago (*111*). The active constituent of foxglove is the cardiac glycoside digoxin, which inhibits Na^+^/K^+^ ATPases in cardiac myocytes, causing an influx of calcium and enhanced muscular contraction. Digoxin has a very narrow therapeutic window, requiring careful monitoring to avoid toxicity. More than 10% of patients taking digoxin excrete high levels of dihydrodigoxin, an inactivemetabolite derived from reduction of an α,β-unsaturated lactone (Fig. 1A). Early studies implicated the gut microbiota in drug inactivation, as coadministering digoxin with antibiotics decreased or abolished dihydrodigoxin production (*112*). Moreover, fecal samples from dihydrodigoxin-excreting individuals were found to completely metabolize digoxin. Subsequent isolation of digoxin-metabolizing microbes revealed that a single organism, *Eubacterium lentum* (renamed *Eggerthella lenta*), was responsible (*113*). However, *E. lenta* was also found in patients who did not excrete dihydrodigoxin, illustrating that the presence of this species alone was not predictive of activity.

Although *E. lenta*’*s* role in digoxinmetabolism has been appreciated for decades, challenges in growing the organism and a lack of genetic tools hampered efforts to understand this transformation. More recently, Turnbaugh and co-workers used RNA sequencing (RNA-seq) and comparative genomics to identify a digoxin-inducible gene cluster present only in digoxin-metabolizing *E. lenta* strains (*37*) (Fig. 1B). The cardiac glycoside reductase (*cgr*) operon fromthis organism encodes two proteins that resemble bacterial reductases involved in anaerobic respiration. Bioinformatic analyses suggest that a membrane-associated cytochrome (Cgr1) transfers electrons through a series of hemes to a predicted flavin-dependent reductase (Cgr2) that converts digoxin to dihydrodigoxin. The presence of *cgr* genes correlates with digoxin reduction by *E. lenta* strains. Furthermore, experiments in GF mice monoassociated with *E. lenta* strains and incubations with human fecal samples indicate that these genes could be useful biomarkers for digoxin inactivation (*37*). Consequently, knowledge of the conditions influencing *cgr* gene expression successfully informed the design of a dietary intervention that reduces digoxin metabolism in vivo (*37*). Overall, this work delineated an approach that may be generalized to study additional inducible microbial metabolic activities. Although *E. lenta*’*s* role in digoxinmetabolism has been appreciated for decades, challenges in growing the organism and a lack of genetic tools hampered efforts to understand this transformation. More recently, Turnbaugh and co-workers used RNA sequencing (RNA-seq) and comparative genomics to identify a digoxin-inducible gene cluster present only in digoxin-metabolizing *E. lenta* strains (*37*) (Fig. 1B). The cardiac glycoside reductase (*cgr*) operon fromthis organism encodes two proteins that resemble bacterial reductases involved in anaerobic respiration. Bioinformatic analyses suggest that a membrane-associated cytochrome (Cgr1) transfers electrons through a series of hemes to a predicted flavin-dependent reductase (Cgr2) that converts digoxin to dihydrodigoxin. The presence of *cgr* genes correlates with digoxin reduction by *E. lenta* strains. Furthermore, experiments in GF mice monoassociated with *E. lenta* strains and incubations with human fecal samples indicate that these genes could be useful biomarkers for digoxin inactivation (*37*). Consequently, knowledge of the conditions influencing *cgr* gene expression successfully informed the design of a dietary intervention that reduces digoxin metabolism in vivo (*37*). Overall, this work delineated an approach that may be generalized to study additional inducible microbial metabolic activities.

### Choline: Uncovering a broadly distributed disease-associated activity

**Fig. 2:**
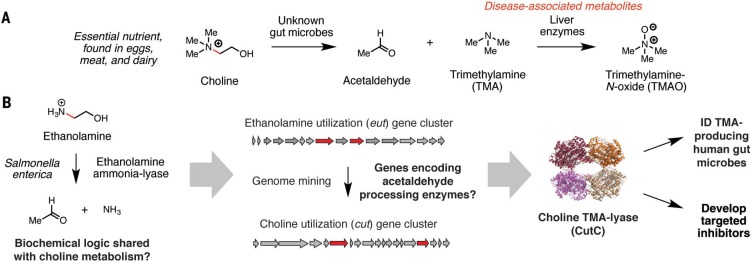
**Uncovering gut microbial enzymes that convert dietary choline to disease-associated metabolites. (A)** Choline is metabolized by a gut microbial-human co-metabolic pathway into the disease-associated metabolites trimethylamine (TMA) and trimethylamine *N*-oxide (TMAO). **(B)** A chemically guided, rational genome-mining effort enabled the identification and characterization of enzymes involved in gut microbial anaerobic choline metabolism.

Gut microbes anaerobically convert choline, an essential nutrient found in meat, eggs, and milk, into TMA, which is further transformed into trimethylamine *N*-oxide (TMAO) by FMOs in the liver (Fig. 2A). This co-metabolic pathway is implicated in several human diseases, including cardiovascular disease (*58*, *59*). A chemically guided, rational genome-mining effort ultimately identified the genes and enzymes that mediate this process (Fig. 2B) (*114*). Choline fermentation begins with a C–N bond cleavage reaction that resembles the first step in ethanolamine utilization, a transformation catalyzed by a B_12_-dependent radical enzyme (ethanolamine ammonia-lyase). Hypothesizing that these pathways might share certain reactions, we searched the genome of the choline-metabolizing, animal-associated strain *Desulfovibrio desulfuricans* for homologs of known ethanolamine-degrading enzymes from the pathogen *Salmonella enterica*. This analysis revealed the choline utilization (*cut*) gene cluster and CutC, a GRE that converts choline into TMA and acetaldehyde (*46*, *114*, *115*).

Biochemical and structural studies have revealed CutC active-site residues that interact with the trimethylammonium group of choline and mediate C–N bond cleavage (*46*, *115*). These conserved amino acids are specific to this particular GRE and have enabled accurate identification of the *cut* pathway in numerous, phylogenetically diverse human gut bacteria (*14*). The discovery of CutC has aided interventions to modulate choline metabolism in vivo, including the design of gut communities with decreased TMA production (*116*) and small-molecule inhibitors targeting this pathway (*117*). Such inhibitors could be potential therapeutics to treat diseases linked to TMA and TMAO production.

### Irinotecan: Inhibiting microbiota-mediated toxicity

**Fig. 3:**
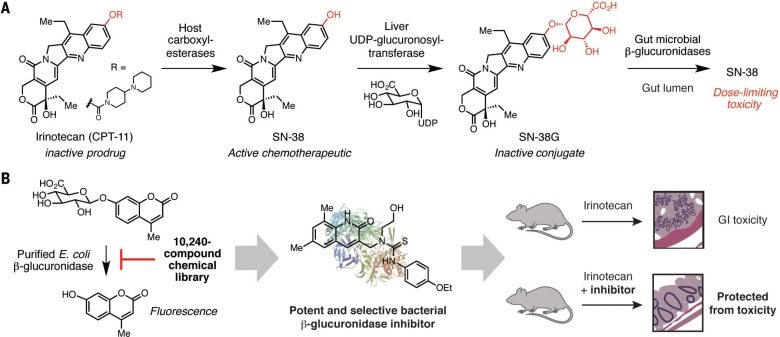
**Preventing drug reactivation and toxicity by inhibiting gut microbial enzymes. (A)** Microbial cleavage of the glucuronidated drug conjugate of the cancer chemotherapeutic SN-38 leads to drug reactivation and toxicity within the gut. UDP, uridine diphosphate. **(B)** High-throughput screening identified specific inhibitors of bacterial β-glucuronidases. These compounds alleviated the GI toxicity associated with irinotecan metabolism. Et, ethyl.

Irinotecan (CPT-11) is a prodrug of SN-38, a topoisomerase inhibitor used for treating cancer (Fig. 3A). SN-38 is glucuronidated by host liver enzymes into an inactive conjugate (SN-38G), which enters the gut via biliary excretion. Gut bacterial β-glucuronidase enzymes hydrolyze SN-38G in the large intestine to regenerate the active chemotherapeutic agent (*26*). SN-38 then enters colonic epithelial cells, causing intestinal damage and severe diarrhea, side effects that limit the use of this otherwise effective drug.

Inhibition of gut bacterial β-glucuronidases is an intriguing approach for preventing drug reactivation. As these enzymes are broadly distributed in commensal bacteria and are present in humans, inhibitors need to be selective for bacterial β-glucuronidases and nontoxic to both host cells and other gut microbes. Redinbo and coworkers have used an in vitro high-throughput screen to successfully identify potent and selective inhibitors of gut bacterial β-glucuronidases (Fig. 3B) (*26*). These inhibitors were effective against multiple, distantly related gut bacteria but did not target the human b-glucuronidase. Structural studies revealed that these compounds interact with an active-site loop distinct to bacterial β-glucuronidases, which explains their selectivity. Administration of one of these inhibitors to mice prevented reactivation of SN-38 in the gut and concomitant toxicity. Further work examining the crystal structures and activities of additional gut bacterial β-glucuronidases identified a conserved Asn-Lysmotif that interacts with the carboxylic acid of the glucuronic acid sugar. This motif is absent from glycosidases that accept different substrates, which will help to identify these enzymes in sequencing data sets and elucidate the effects of inhibitors on different types of gut organisms (*23*). Because bacterial β-glucuronidases can deconjugate glucuronides derived from many dietary compounds and drugs, inhibitors of these enzymesmay be useful in other therapeutic contexts (*27*). By modulating specific metabolic activities, additional small-molecule inhibitors of microbial gut enzymes may help to uncover the roles of particular transformations in this complex habitat and could be excellent starting points for developingmicrobiotatargeted drugs.

## Outlook and challenges

Although our appreciation of the human gut microbiota’s role in transforming xenobiotics has increased, our understanding of the biological implications of these reactions is limited. The future challenges lie in identifying the organisms, genes, and enzymes involved in known metabolic processes; uncovering important but currently unappreciated activities; and elucidating the effects of this chemistry on both host and microbiota.

### Connecting xenobiotic metabolism to organisms, genes, and enzymes

As we have discussed above, most examples of gut microbial xenobiotic metabolism are associated with the whole gut community. Surveys of human gut isolates, such as Human Microbiome Project (HMP) reference strains (*118*), or culturing from human fecal samples can identify individual organisms with particular metabolic capabilities. Examining intact communities via approaches like stable isotope probing, fluorescence in situ hybridization, and imaging mass spectrometry can also be used to detect individual cells that metabolize particular compounds (*119*). Coupled with single-cell genomics, these methods may aid investigations of activities associated with organisms that are challenging to cultivate (*120*).

Both traditional approaches (chemical or transposon mutagenesis and activity-guided protein purification) and more recent strategies (rational genome mining, comparative genomics, RNA-seq, and functionalmetagenomics) may be used to link metabolic activities with genes (*37*, *114*, *121*). However, a lack of genetic tools for many gut microbes and the narrow range of heterologous hosts available for functional characterization still limit progress toward this objective. An appreciation of the chemical reactivity required for xenobiotic degradation is vital for informing genome and metagenome mining, as well as interpreting and rationalizing results from othermethods.Microbes in which enzymes and metabolic pathways are better studied, including pathogens and environmental strains involved in bioremediation, may also offer clues to support microbiome-focused discovery efforts.

The examples of xenobiotic metabolism surveyed above likely represent a small fraction of the transformations taking place in the gut community. Metabolomics in human subjects will play a key role in illuminating currently unappreciated gut microbial metabolic processes, particularly for transformations that depend on additional microbial and host activities or interactions (*122*).

### Linking uncharacterized genes in microbiomes to xenobiotic metabolism

We should expect to uncover interesting gut microbial activities by investigating the vast number of uncharacterized or misannotated genes present in human gut microbiomes. Notably, 86% of the genes from HMP stool metagenomes cannot be assigned to known metabolic pathways, and half cannot be annotated (*10*). Many members of large enzyme superfamilies also have unknown functions and are typically misannotated. Collaborative efforts such as the Enzyme Function Initiative have advanced bioinformatic and experimental approaches for functionally characterizing such enzymes (*123*), including protein sequence similarity and genome neighborhood network analyses, high-throughput ligand docking, and structural genomics. These methods highlight divergent sequences that likely possess distinct functions and provide context for linking uncharacterized enzymes withmetabolic pathways and substrates. Conversely, an understanding of the chemistry involved in known xenobioticmetabolism may pinpoint particular enzyme superfamilies as starting points to prospect for additional transformations.

Comprehensively characterizing proteins that lack homology to known enzymes presents amore substantial challenge and will likely require novel approaches that incorporate automation and highthroughput assay formats. Meanwhile, it is critical to prioritize uncharacterized proteins for further study by considering both ecological context (i.e., abundance and distribution in the gut microbiome) and host health status, including exposure to xenobiotics (*16*, *124*).

### Harnessing knowledge of microbial metabolism to improve human health

**Fig. 4:**
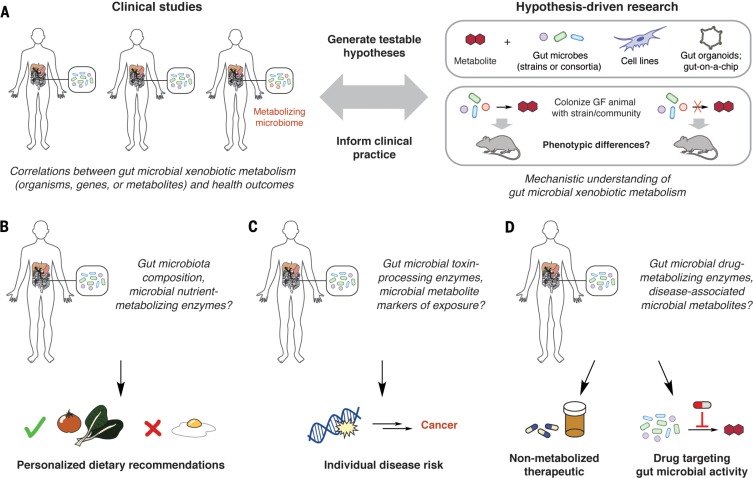
**Potential implications of understanding gut microbial xenobiotic metabolism. (A)** Interfacing clinical studies and hypothesis-driven research in model systems is essential for elucidating the biological consequences of gut microbial xenobiotic metabolism. Incorporating a mechanistic understanding of microbial transformations, along with knowledge of host genetics and metabolism, could **(B)** inform personalized nutrition, **(C)** improve toxicological risk assessment, and **(D)** enable personalized medicine.

Deciphering how gut microbial transformation of xenobiotics affects host health will require the integration of clinical studies with mechanistic experiments in model systems and organisms (Fig. 4A). These efforts will necessitate identifying microbial genes and/or metabolites that are reliable, diagnostic markers for activities of interest. Obtaining data from patients is particularly important, and existing epidemiological studies linking diet or xenobiotic exposure to health outcomes should be reexamined with gut microbial participation in mind.

Diet is clearly a cornerstone of human health. Though many epidemiological studies linking dietary patterns with health outcomes yield conflicting results, few have taken gut microbial metabolic capabilities into account. A comprehensive molecular understanding of how gut microbes process dietary components is essential for the rational use of “functional foods” or prebiotics to treat conditions such as metabolic disease and malnutrition. This knowledge can also inform personalized nutrition, in which diets are individually customized for patients’ metabolic profiles and gut microbiotas (Fig. 4B) (*125*).

Similarly to dietary studies, attempts to correlate pollutant exposure with health outcomes often yield conflicting results that could, in part, arise from variations inmicrobial metabolic activities (*126*).Gut microbial enzymes that alter the toxicity of industrial chemicals and environmental pollutants could serve as biomarkers to inform risk assessments among populations exposed to these compounds (Fig. 4C). It may also become possible to use gut microbial metabolism to help remove harmful compounds from the body and prevent disease, analogous to bioremediation of polluted environments (*127*).

Finally, knowledge of how gut microbes transformpharmaceuticals should be further integrated into drug development, clinical trial design, and clinical practice. Understanding which functional groups are prone to microbial metabolism allows medicinal chemists to either avoid these structural features or incorporate them into prodrugs to enable selective activation in the GI tract (*38*, *39*). Gut microbial enzymes that mediate harmful metabolic activities may also represent a new class of drug targets. Finally, knowledge of gutmicrobial metabolism could shape clinical trial design and clinical practice by allowing physicians to screen for detrimental or beneficial activities before prescribing drugs (Fig. 4D). Ultimately, personalized medicine will require a better understanding of the distribution of specific microbial metabolic functions within human populations.

## Conclusions

A major challenge facing human gut microbiota research is the need to move beyond cataloging the organisms and genes in this community to elucidating the mechanisms underlying their influence on host health. By altering the chemical structures of ingested compounds, gut microbes can mediate the effects of diet, pollutants, and drugs on host physiology. Individual variation remains a major challenge, and although many such metabolic activities have been identified, few have been connected to organisms, genes, and enzymes.Moving forward, it is essential that we incorporate enzyme discovery and characterization efforts into investigations of gut microbial xenobiotic metabolism. Only by gaining a molecular understanding of these processes can we leverage the remarkable chemical abilities of this community to improve human health.

## References

[1] Moeller A. H. et al., Cospeciation of gut microbiota with hominids. *Science* 353, 380–382 (2016). doi: 10.1126/science.aaf3951; pmid: 27463672 PMC4995445

[2] Buffie C. G. et al., Precision microbiome reconstitution restores bile acid mediated resistance to *Clostridium difficile*. *Nature* 517, 205–208 (2015). doi: 10.1038/nature13828; pmid: 25337874 PMC4354891

[3] Sharon G. et al., Specialized metabolites from the microbiome in health and disease. *Cell Metab*. 20, 719–730 (2014). doi: 10.1016/j.cmet.2014.10.016; pmid: 25440054 PMC4337795

[4] Koh A., De Vadder F., Kovatcheva-Datchary P., Bäckhed F., From dietary fiber to host physiology: Short-chain fatty acids as key bacterial metabolites. *Cell* 165, 1332–1345 (2016). doi: 10.1016/j.cell.2016.05.041; pmid: 27259147

[5] Sousa T. et al., The gastrointestinal microbiota as a site for the biotransformation of drugs. *Int. J. Pharm*. 363, 1–25 (2008). doi: 10.1016/j.ijpharm.2008.07.009; pmid: 18682282

[6] Danielsson H., Gustafsson B., On serum-cholesterol levels and neutral fecal sterols in germ-free rats; bile acids and steroids 59. *Arch. Biochem. Biophys*. 83, 482–485 (1959). doi: 10.1016/0003-9861(59)90056-6; pmid: 13813997

[7] Sender R., Fuchs S., Milo R., Revised estimates for the number of human and bacteria cells in the body. *PLOS Biol*. 14, e1002533 (2016). doi: 10.1371/journal.pbio.1002533; pmid: 27541692 PMC4991899

[8] Aron-Wisnewsky J., Doré J., Clement K., The importance of the gut microbiota after bariatric surgery. *Nat. Rev. Gastroenterol. Hepatol*. 9, 590–598 (2012). doi: 10.1038/nrgastro.2012.161; pmid: 22926153

[9] Eckburg P. B. et al., Diversity of the human intestinal microbial flora. *Science* 308, 1635–1638 (2005). doi: 10.1126/science.1110591; pmid: 15831718 PMC1395357

[10] The Human Microbiome Project Consortium, Structure, function and diversity of the healthy human microbiome. *Nature* 486, 207–214 (2012). doi: 10.1038/nature11234; pmid: 22699609 PMC3564958

[11] Wang B., Hu L., Siahaan T., *Drug Delivery: Principles and Applications* (Wiley, ed. 2, 2016).

[12] Linhardt R. J., Galliher P. M., Cooney C. L., Polysaccharide lyases. *Appl. Biochem. Biotechnol*. 12, 135–176 (1987). doi: 10.1007/BF02798420; pmid: 3521491

[13] Ryan A. et al., Identification of NAD(P)H quinone oxidoreductase activity in azoreductases from *P. aeruginosa*: Azoreductases and NAD(P)H quinone oxidoreductases belong to the same FMN-dependent superfamily of enzymes. *PLOS ONE* 9, e98551 (2014). doi: 10.1371/journal.pone.0098551; pmid: 24915188 PMC4051601

[14] Martínez-del Campo A. et al., Characterization and detection of a widely distributed gene cluster that predicts anaerobic choline utilization by human gut bacteria. *mBio* 6, e00042-15 (2015). doi: 10.1128/mBio.00042-15; pmid: 25873372 PMC4453576

[15] El Kaoutari A., Armougom F., Gordon J. I., Raoult D., Henrissat B., The abundance and variety of carbohydrateactive enzymes in the human gut microbiota. *Nat. Rev. Microbiol*. 11, 497–504 (2013). doi: 10.1038/nrmicro3050; pmid: 23748339

[16] Levin B. J. et al., A prominent glycyl radical enzyme in human gut microbiomes metabolizes *trans*-4-hydroxy-L-proline. *Science* 355, eaai8386 (2017). doi: 10.1126/science.aai8386; pmid: 28183913 PMC5705181

[17] Kurokawa K. et al., Comparative metagenomics revealed commonly enriched gene sets in human gut microbiomes. *DNA Res*. 14, 169–181 (2007). doi: 10.1093/dnares/dsm018; pmid: 17916580 PMC2533590

[18] Ellrott K., Jaroszewski L., Li W., Wooley J. C., Godzik A., Expansion of the protein repertoire in newly explored environments: Human gut microbiome specific protein families. *PLOS Comput. Biol*. 6, e1000798 (2010). doi: 10.1371/journal.pcbi.1000798; pmid: 20532204 PMC2880560

[19] Jancova P., Anzenbacher P., Anzenbacherova E., Phase II drug metabolizing enzymes. *Biomed. Pap. Med. Fac. Univ. Palacky Olomouc Czech Repub*. 154, 103–116 (2010). doi: 10.5507/bp.2010.017; pmid: 20668491

[20] Wang J., Yadav V., Smart A. L., Tajiri S., Basit A. W., Stability of peptide drugs in the colon. *Eur. J. Pharm. Sci*. 78, 31–36 (2015). doi: 10.1016/j.ejps.2015.06.018; pmid: 26111980

[21] Tozaki H. et al., Degradation of insulin and calcitonin and their protection by various protease inhibitors in rat caecal contents: Implications in peptide delivery to the colon. *J. Pharm. Pharmacol*. 49, 164–168 (1997). doi: 10.1111/j.2042-7158.1997.tb06773.x; pmid: 9055189

[22] Kallemeijn W. W., Witte M. D., Wennekes T., Aerts J. M., Mechanism-based inhibitors of glycosidases: Design and applications. *Adv. Carbohydr. Chem. Biochem*. 71, 297–338 (2014). doi: 10.1016/B978-0-12-800128-8.00004-2; pmid: 25480507

[23] Wallace B. D. et al., Structure and inhibition of microbiome β-glucuronidases essential to the alleviation of cancer drug toxicity. *Chem. Biol*. 22, 1238–1249 (2015). doi: 10.1016/j.chembiol.2015.08.005; pmid: 26364932 PMC4575908

[24] Ulmer J. E. et al., Characterization of glycosaminoglycan (GAG) sulfatases from the human gut symbiont *Bacteroides thetaiotaomicron* reveals the first GAG-specific bacterial endosulfatase. *J. Biol. Chem*. 289, 24289–24303 (2014). doi: 10.1074/jbc.M114.573303; pmid: 25002587 PMC4148858

[25] Lukatela G. et al., Crystal structure of human arylsulfatase A: The aldehyde function and the metal ion at the active site suggest a novel mechanism for sulfate ester hydrolysis. *Biochemistry* 37, 3654–3664 (1998). doi: 10.1021/bi9714924; pmid: 9521684

[26] Wallace B. D. et al., Alleviating cancer drug toxicity by inhibiting a bacterial enzyme. *Science* 330, 831–835 (2010). doi: 10.1126/science.1191175; pmid: 21051639 PMC3110694

[27] Saitta K. S. et al., Bacterial b-glucuronidase inhibition protects mice against enteropathy induced by indomethacin, ketoprofen or diclofenac: Mode of action and pharmacokinetics. *Xenobiotica* 44, 28–35 (2014). doi: 10.3109/00498254.2013.811314; pmid: 23829165 PMC3972617

[28] Carter J. H., McLafferty M. A., Goldman P., Role of the gastrointestinal microflora in amygdalin (laetrile)-induced cyanide toxicity. *Biochem. Pharmacol*. 29, 301–304 (1980). doi: 10.1016/0006-2952(80)90504-3; pmid: 7362642

[29] Nimura T., Tokieda T., Yamaha T., Partial purification and some properties of cyclamate sulfamatase. *J. Biochem*. 75, 407–417 (1974). doi: 10.1093/oxfordjournals.jbchem.a130407; pmid: 4209783

[30] Donohoe D. R. et al., The microbiome and butyrate regulate energy metabolism and autophagy in the mammalian colon. *Cell Metab*. 13, 517–526 (2011). doi: 10.1016/j.cmet.2011.02.018; pmid: 21531334 PMC3099420

[31] Cooper A. J. et al., Cysteine *S*-conjugate β-lyases: Important roles in the metabolism of naturally occurring sulfur and selenium-containing compounds, xenobiotics and anticancer agents. *Amino Acids* 41, 7–27 (2011). doi: 10.1007/s00726-010-0552-0; pmid: 20306345 PMC2898922

[32] Claus S. P., Guillou H., Ellero-Simatos S., The gut microbiota: A major player in the toxicity of environmental pollutants? *NPJ Biofilms Microbiomes* 2, 16003 (2016). doi: 10.1038/npjbiofilms.2016.3 28721242 PMC5515271

[33] Rossol I., Pühler A., The *Corynebacterium glutamicum aecD* gene encodes a C-S lyase with α,β-elimination activity that degrades aminoethylcysteine. *J. Bacteriol*. 174, 2968–2977 (1992). doi: 10.1128/jb.174.9.2968-2977.1992; pmid: 1569026 PMC205951

[34] Rafii F., Hall J. D., Cerniglia C. E., Mutagenicity of azo dyes used in foods, drugs and cosmetics before and after reduction by *Clostridium* species from the human intestinal tract. *Food Chem. Toxicol*. 35, 897–901 (1997). doi: 10.1016/S0278-6915(97)00060-4; pmid: 9409630

[35] Lee S. C., Renwick A. G., Sulphoxide reduction by rat intestinal flora and by *Escherichia coli* in vitro. *Biochem. Pharmacol*. 49, 1567–1576 (1995). doi: 10.1016/0006-2952(95)00093-F; pmid: 7786297

[36] Laue H., Friedrich M., Ruff J., Cook A. M., Dissimilatory sulfite reductase (desulfoviridin) of the taurine-degrading, nonsulfate- reducing bacterium *Bilophila wadsworthia* RZATAU contains a fused DsrB-DsrD subunit. *J. Bacteriol*. 183, 1727–1733 (2001). doi: 10.1128/JB.183.5.1727-1733.2001; pmid: 11160104 PMC95058

[37] Haiser H. J. et al., Predicting and manipulating cardiac drug inactivation by the human gut bacterium *Eggerthella lenta*. *Science* 341, 295–298 (2013). doi: 10.1126/science.1235872; pmid: 23869020 PMC3736355

[38] Peppercorn M. A., Goldman P., The role of intestinal bacteria in the metabolism of salicylazosulfapyridine. *J. Pharmacol. Exp. Ther*. 181, 555–562 (1972). pmid: 4402374

[39] Lavrijsen K. et al., Reduction of the prodrug loperamide oxide to its active drug loperamide in the gut of rats, dogs, and humans. *Drug Metab. Dispos*. 23, 354–362 (1995). pmid: 7628301

[40] Rafii F., Cerniglia C. E., Reduction of azo dyes and nitroaromatic compounds by bacterial enzymes from the human intestinal tract. *Environ. Health Perspect*. 103 (suppl. 5), 17–19 (1995). doi: 10.1289/ehp.95103s417; pmid: 8565901 PMC1519296

[41] Kumano T., Fujiki E., Hashimoto Y., Kobayashi M., Discovery of a sesamin-metabolizing microorganism and a new enzyme. *Proc. Natl. Acad. Sci. U.S.A*. 113, 9087–9092 (2016). doi: 10.1073/pnas.1605050113; pmid: 27444012 PMC4987775

[42] Ticak T., Kountz D. J., Girosky K. E., Krzycki J. A., Ferguson D. J. Jr., A nonpyrrolysine member of the widely distributed trimethylamine methyltransferase family is a glycine betaine methyltransferase. *Proc. Natl. Acad. Sci. U.S.A*. 111, E4668–E4676 (2014). doi: 10.1073/pnas.1409642111; pmid: 25313086 PMC4217433

[43] Deloménie C. et al., Identification and functional characterization of arylamine *N*-acetyltransferases in eubacteria: Evidence for highly selective acetylation of 5- aminosalicylic acid. *J. Bacteriol*. 183, 3417–3427 (2001). doi: 10.1128/JB.183.11.3417-3427.2001; pmid: 11344150 PMC99640

[44] Sutton D., Butler A. M., Nadin L., Murray M., Role of CYP3A4 in human hepatic diltiazem *N*-demethylation: Inhibition of CYP3A4 activity by oxidized diltiazem metabolites. *J. Pharmacol. Exp. Ther*. 282, 294–300 (1997). pmid: 9223567

[45] Buckel W., Golding B. T., Radical enzymes in anaerobes. *Annu. Rev. Microbiol*. 60, 27–49 (2006). doi: 10.1146/annurev.micro.60.080805.142216; pmid: 16704345

[46] Bodea S., Funk M. A., Balskus E. P., Drennan C. L., Molecular basis of C–N bond cleavage by the glycyl radical enzyme choline trimethylamine-lyase. *Cell Chem. Biol*. 23, 1206–1216 (2016). doi: 10.1016/j.chembiol.2016.07.020; pmid: 27642068 PMC5493019

[47] Selmer T., Andrei P. I., *p*-Hydroxyphenylacetate decarboxylase from *Clostridium difficile*. *Eur. J. Biochem*. 268, 1363–1372 (2001). doi: 10.1046/j.1432-1327.2001.02001.x; pmid: 11231288

[48] Clayton T. A., Baker D., Lindon J. C., Everett J. R., Nicholson J. K., Pharmacometabonomic identification of a significant host-microbiome metabolic interaction affecting human drug metabolism. *Proc. Natl. Acad. Sci. U.S.A*. 106, 14728–14733 (2009). doi: 10.1073/pnas.0904489106; pmid: 19667173 PMC2731842

[49] He X., Marco M. L., Slupsky C. M., Emerging aspects of food and nutrition on gut microbiota. *J. Agric. Food Chem*. 61, 9559–9574 (2013). doi: 10.1021/jf4029046; pmid: 24028159

[50] Flint H. J., Bayer E. A., Rincon M. T., Lamed R., White B. A., Polysaccharide utilization by gut bacteria: Potential for new insights from genomic analysis. *Nat. Rev. Microbiol*. 6, 121–131 (2008). doi: 10.1038/nrmicro1817; pmid: 18180751

[51] Koropatkin N. M., Cameron E. A., Martens E. C., How glycan metabolism shapes the human gut microbiota. *Nat. Rev. Microbiol*. 10, 323–335 (2012). doi: 10.1038/nrmicro2746; pmid: 22491358 PMC4005082

[52] Caminero A. et al., Differences in gluten metabolism among healthy volunteers, coeliac disease patients and first-degree relatives. *Br. J. Nutr*. 114, 1157–1167 (2015). doi: 10.1017/S0007114515002767; pmid: 26428276

[53] Caminero A. et al., Duodenal bacteria from patients with celiac disease and healthy subjects distinctly affect gluten breakdown and immunogenicity. *Gastroenterology* 151, 670–683 (2016). doi: 10.1053/j.gastro.2016.06.041; pmid: 27373514

[54] Blachier F., Mariotti F., Huneau J. F., Tomé D., Effects of amino acid-derived luminal metabolites on the colonic epithelium and physiopathological consequences. *Amino Acids* 33, 547–562 (2007). doi: 10.1007/s00726-006-0477-9; pmid: 17146590

[55] Wikoff W. R. et al., Metabolomics analysis reveals large effects of gut microflora on mammalian blood metabolites. *Proc. Natl. Acad. Sci. U.S.A*. 106, 3698–3703 (2009). doi: 10.1073/pnas.0812874106; pmid: 19234110 PMC2656143

[56] Williams B. B. et al., Discovery and characterization of gut microbiota decarboxylases that can produce the neurotransmitter tryptamine. *Cell Host Microbe* 16, 495–503 (2014). doi: 10.1016/j.chom.2014.09.001; pmid: 25263219 PMC4260654

[57] Devlin A. S. et al., Modulation of a circulating uremic Solute via rational genetic manipulation of the gut microbiota. *Cell Host Microbe* 20, 709–715 (2016). doi: 10.1016/j.chom.2016.10.021; pmid: 27916477 PMC5159218

[58] Cho C. E., Caudill M. A., Trimethylamine-*N*-oxide: Friend, foe, or simply caught in the cross-fire? *Trends Endocrinol. Metab*. 28, 121–130 (2017). doi: 10.1016/j.tem.2016.10.005; pmid: 27825547

[59] Fennema D., Phillips I. R., Shephard E. A., Trimethylamine and trimethylamine *N*-oxide, a flavin-containing monooxygenase 3 (FMO3)-mediated host-microbiome metabolic axis implicated in health and disease. *Drug Metab. Dispos*. 44, 1839–1850 (2016). doi: 10.1124/dmd.116.070615; pmid: 27190056 PMC5074467

[60] Escolà-Gil J. C. et al., The cholesterol content of Western diets plays a major role in the paradoxical increase in highdensity lipoprotein cholesterol and upregulates the macrophage reverse cholesterol transport pathway. *Arterioscler. Thromb. Vasc. Biol*. 31, 2493–2499 (2011). doi: 10.1161/ATVBAHA.111.236075; pmid: 21885848

[61] Macdonald I. A., Bokkenheuser V. D., Winter J., McLernon A. M., Mosbach E. H., Degradation of steroids in the human gut. *J. Lipid Res*. 24, 675–700 (1983). pmid: 6350517

[62] Gérard P. et al., Gnotobiotic rats harboring human intestinal microbiota as a model for studying cholesterol-tocoprostanol conversion. *FEMS Microbiol. Ecol*. 47, 337–343 (2004). doi: 10.1016/S0168-6496(03)00285-X; pmid: 19712322

[63] Ren D., Li L., Schwabacher A. W., Young J. W., Beitz D. C., Mechanism of cholesterol reduction to coprostanol by *Eubacterium coprostanoligenes* ATCC 51222. *Steroids* 61, 33–40 (1996). doi: 10.1016/0039-128X(95)00173-N; pmid: 8789734

[64] Dujovne C. A. et al., Efficacy and safety of a potent new selective cholesterol absorption inhibitor, ezetimibe, in patients with primary hypercholesterolemia. *Am. J. Cardiol*. 90, 1092–1097 (2002). doi: 10.1016/S0002-9149(02)02798-4; pmid: 12423709

[65] Valdés L. et al., The relationship between phenolic compounds from diet and microbiota: Impact on human health. *Food Funct*. 6, 2424–2439 (2015). doi: 10.1039/C5FO00322A; pmid: 26068710

[66] García-Villalba R., Beltrán D., Espín J. C., Selma M. V., Tomás-Barberán F. A., Time course production of urolithins from ellagic acid by human gut microbiota. *J. Agric. Food Chem*. 61, 8797–8806 (2013). doi: 10.1021/jf402498b; pmid: 23984796

[67] Atkinson C., Frankenfeld C. L., Lampe J. W., Gut bacterial metabolism of the soy isoflavone daidzein: Exploring the relevance to human health. *Exp. Biol. Med*. 230, 155–170 (2005). pmid: 15734719 10.1177/153537020523000302

[68] Tang J. et al., Tea consumption and mortality of all cancers, CVD and all causes: A meta-analysis of eighteen prospective cohort studies. *Br. J. Nutr*. 114, 673–683 (2015). doi: 10.1017/S0007114515002329; pmid: 26202661

[69] Clavel T., Henderson G., Engst W., Doré J., Blaut M., Phylogeny of human intestinal bacteria that activate the dietary lignan secoisolariciresinol diglucoside. *FEMS Microbiol. Ecol*. 55, 471–478 (2006). doi: 10.1111/j.1574-6941.2005.00057.x; pmid: 16466386

[70] Bopp B. A., Sonders R. C., Kesterson J. W., Renwick A. G., Toxicological aspects of cyclamate and cyclohexylamine. *Crit. Rev. Toxicol*. 16, 213–306 (1986). doi: 10.3109/10408448609037465; pmid: 2420530

[71] Renwick A. G., Tarka S. M., Microbial hydrolysis of steviol glycosides. *Food Chem. Toxicol*. 46 (suppl. 7), S70–S74 (2008). doi: 10.1016/j.fct.2008.05.008; pmid: 18550247

[72] Krishnan R. et al., The effect of dietary xylitol on the ability of rat caecal flora to metabolise xylitol. *Aust. J. Exp. Biol. Med. Sci*. 58, 639–652 (1980). doi: 10.1038/icb.1980.66; pmid: 6791623

[73] Renwick A. G., The metabolism of intense sweeteners. *Xenobiotica* 16, 1057–1071 (1986). doi: 10.3109/00498258609038983; pmid: 3541395

[74] Kassie F. et al., Intestinal microflora plays a crucial role in the genotoxicity of the cooked food mutagen 2-amino-3- methylimidazo [4,5-*f*]quinoline. *Carcinogenesis* 22, 1721–1725 (2001). doi: 10.1093/carcin/22.10.1721; pmid: 11577015

[75] Humblot C. et al., β-glucuronidase in human intestinal microbiota is necessary for the colonic genotoxicity of the food-borne carcinogen 2-amino-3-methylimidazo[4,5-*f*] quinoline in rats. *Carcinogenesis* 28, 2419–2425 (2007). doi: 10.1093/carcin/bgm170; pmid: 17660508

[76] Cross A. J. et al., A large prospective study of meat consumption and colorectal cancer risk: An investigation of potential mechanisms underlying this association. *Cancer Res*. 70, 2406–2414 (2010). doi: 10.1158/0008-5472.CAN-09-3929; pmid: 20215514 PMC2840051

[77] Rafii F., Franklin W., Cerniglia C. E., Azoreductase activity of anaerobic bacteria isolated from human intestinal microflora. *Appl. Environ. Microbiol*. 56, 2146–2151 (1990). pmid: 2202258 10.1128/aem.56.7.2146-2151.1990 PMC184574

[78] Borzelleca J. F., Depukat K., Hallagan J. B., Lifetime toxicity/carcinogenicity studies of FD & C blue no. 1 (brilliant blue FCF) in rats and mice. *Food Chem. Toxicol*. 28, 221–234 (1990). doi: 10.1016/0278-6915(90)90034-K; pmid: 2358248

[79] Singh Z., Chadha P., Textile industry and occupational cancer. *J. Occup. Med. Toxicol*. 11, 39 (2016). doi: 10.1186/s12995-016-0128-3; pmid: 27532013 PMC4986180

[80] Bos R. P. et al., Internal exposure of rats to benzidine derived from orally administered benzidine-based dyes after intestinal azo reduction. *Toxicology* 40, 207–213 (1986). doi: 10.1016/0300-483X(86)90080-6; pmid: 3726894

[81] Ingelfinger J. R., Melamine and the global implications of food contamination. *N. Engl. J. Med*. 359, 2745–2748 (2008). doi: 10.1056/NEJMp0808410; pmid: 19109571

[82] Zheng X. et al., Melamine-induced renal toxicity is mediated by the gut microbiota. *Sci. Transl. Med*. 5, 172ra22 (2013). doi: 10.1126/scitranslmed.3005114; pmid: 23408055

[83] Wang H., Geng C., Li J., Hu A., Yu C. P., Characterization of a novel melamine-degrading bacterium isolated from a melamine-manufacturing factory in China. *Appl. Microbiol. Biotechnol*. 98, 3287–3293 (2014). doi: 10.1007/s00253-013-5363-2; pmid: 24297478

[84] Rowland I. R., Davies M. J., Grasso P., Metabolism of methylmercuric chloride by the gastro-intestinal flora of the rat. *Xenobiotica* 8, 37–43 (1978). doi: 10.3109/00498257809060381; pmid: 626001

[85] Rowland I. R., Davies M. J., Evans J. G., The effect of the gastrointestinal flora on tissue content of mercury and organomercurial neurotoxicity in rats given methylmercuric chloride. *Dev. Toxicol. Environ. Sci*. 8, 79–82 (1980). pmid: 7308081

[86] Liebert C. A., Wireman J., Smith T., Summers A. O., Phylogeny of mercury resistance (*mer*) operons of gramnegative bacteria isolated from the fecal flora of primates. *Appl. Environ. Microbiol*. 63, 1066–1076 (1997). pmid: 9055422 10.1128/aem.63.3.1066-1076.1997 PMC168397

[87] Rothenberg S. E. et al., The role of gut microbiota in fetal methylmercury exposure: Insights from a pilot study. *Toxicol. Lett*. 242, 60–67 (2016). doi: 10.1016/j.toxlet.2015.11.022; pmid: 26626101 PMC4707065

[88] Diaz-Bone R. A., van de Wiele T. R., Biovolatilization of metal(loid)s by intestinal microorganisms in the simulator of the human intestinal microbial ecosystem. *Environ. Sci. Technol*. 43, 5249–5256 (2009). doi: 10.1021/es900544c; pmid: 19708349

[89] Spanogiannopoulos P., Bess E. N., Carmody R. N., Turnbaugh P. J., The microbial pharmacists within us: A metagenomic view of xenobiotic metabolism. *Nat. Rev. Microbiol*. 14, 273–287 (2016). doi: 10.1038/nrmicro.2016.17; pmid: 26972811 PMC5243131

[90] Takeno S., Hirano Y., Kitamura A., Sakai T., Comparative developmental toxicity and metabolism of nitrazepam in rats and mice. *Toxicol. Appl. Pharmacol*. 121, 233–238 (1993). doi: 10.1006/taap.1993.1150; pmid: 8346540

[91] Okuda H. et al., Lethal drug interactions of sorivudine, a new antiviral drug, with oral 5-fluorouracil prodrugs. *Drug Metab. Dispos*. 25, 270–273 (1997). pmid: 9152608

[92] Vétizou M. et al., Anticancer immunotherapy by CTLA-4 blockade relies on the gut microbiota. *Science* 350, 1079–1084 (2015). doi: 10.1126/science.aad1329; pmid: 26541610 PMC4721659

[93] Shin N. R. et al., An increase in the *Akkermansia spp*. population induced by metformin treatment improves glucose homeostasis in diet-induced obese mice. *Gut* 63, 727–735 (2014). doi: 10.1136/gutjnl-2012-303839; pmid: 23804561

[94] van Hogezand R. A. et al., Bacterial acetylation of 5- aminosalicylic acid in faecal suspensions cultured under aerobic and anaerobic conditions. *Eur. J. Clin. Pharmacol*. 43, 189–192 (1992). doi: 10.1007/BF01740669; pmid: 1425876

[95] Strong H. A., Renwick A. G., George C. F., Liu Y. F., Hill M. J., The reduction of sulphinpyrazone and sulindac by intestinal bacteria. *Xenobiotica* 17, 685–696 (1987). doi: 10.3109/00498258709043976; pmid: 3630204

[96] Lehouritis P. et al., Local bacteria affect the efficacy of chemotherapeutic drugs. *Sci. Rep*. 5, 14554 (2015). doi: 10.1038/srep14554; pmid: 26416623 PMC4586607

[97] Calne D. B. et al., Idiopathic Parkinsonism treated with an extracerebral decarboxylase inhibitor in combination with levodopa. *BMJ* 3, 729–732 (1971). doi: 10.1136/bmj.3.5777.729; pmid: 4938431 PMC1798919

[98] Bergmark J. et al., Decarboxylation of orally administered L-dopa in the human digestive tract. *Naunyn Schmiedebergs Arch. Pharmacol*. 272, 437–440 (1972). doi: 10.1007/BF00501249; pmid: 4260263

[99] Goldin B. R., Peppercorn M. A., Goldman P., Contributions of host and intestinal microflora in the metabolism of L-dopa by the rat. *J. Pharmacol. Exp. Ther*. 186, 160–166 (1973). pmid: 4723308

[100] Nutt J. G., Holford N. H., The response to levodopa in Parkinson’s disease: Imposing pharmacological law and order. *Ann. Neurol*. 39, 561–573 (1996). doi: 10.1002/ana.410390504; pmid: 8619540

[101] Zhang K., Ni Y., Tyrosine decarboxylase from *Lactobacillus brevis*: Soluble expression and characterization. *Protein Expr. Purif*. 94, 33–39 (2014). doi: 10.1016/j.pep.2013.10.018; pmid: 24211777

[102] Sharon G., Sampson T. R., Geschwind D. H., Mazmanian S. K., The central nervous system and the gut microbiome. *Cell* 167, 915–932 (2016). doi: 10.1016/j.cell.2016.10.027; pmid: 27814521 PMC5127403

[103] Relman A. S., Closing the books on laetrile. *N. Engl. J. Med*. 306, 236 (1982). doi: 10.1056/NEJM198201283060410; pmid: 7054687

[104] Moertel C. G. et al., A clinical trial of amygdalin (laetrile) in the treatment of human cancer. *N. Engl. J. Med*. 306, 201–206 (1982). doi: 10.1056/NEJM198201283060403; pmid: 7033783

[105] Strugala G. J., Rauws A. G., Elbers R., Intestinal first pass metabolism of amygdalin in the rat in vitro. *Biochem. Pharmacol*. 35, 2123–2128 (1986). doi: 10.1016/0006-2952(86)90580-0; pmid: 3089225

[106] Feng R. et al., Transforming berberine into its intestineabsorbable form by the gut microbiota. *Sci. Rep*. 5, 12155 (2015). doi: 10.1038/srep12155; pmid: 26174047 PMC4502414

[107] Liu Y. et al., Ginsenoside metabolites, rather than naturally occurring ginsenosides, lead to inhibition of human cytochrome P450 enzymes. *Toxicol. Sci*. 91, 356–364 (2006). doi: 10.1093/toxsci/kfj164; pmid: 16547074

[108] Konstantinidis K. T., Tiedje J. M., Genomic insights that advance the species definition for prokaryotes. *Proc. Natl. Acad. Sci. U.S.A*. 102, 2567–2572 (2005). doi: 10.1073/pnas.0409727102; pmid: 15701695 PMC549018

[109] Li J. et al., An integrated catalog of reference genes in the human gut microbiome. *Nat. Biotechnol*. 32, 834–841 (2014). doi: 10.1038/nbt.2942; pmid: 24997786

[110] Das A., Srinivasan M., Ghosh T. S., Mande S. S., Xenobiotic metabolism and gut microbiomes. *PLOS ONE* 11, e0163099 (2016). doi: 10.1371/journal.pone.0163099; pmid: 27695034 PMC5047465

[111] Withering W., *An Account of the Foxglove* (printed by M. Swinney for G. G. J. and J. Robinson, London, Birmingham, 1785).

[112] Lindenbaum J., Rund D. G., Butler V. P. Jr., Tse-Eng D., Saha J. R., Inactivation of digoxin by the gut flora: Reversal by antibiotic therapy. *N. Engl. J. Med*. 305, 789–794 (1981). doi: 10.1056/NEJM198110013051403; pmid: 7266632

[113] Saha J. R., Butler V. P. Jr., Neu H. C., Lindenbaum J., Digoxin-inactivating bacteria: Identification in human gut flora. *Science* 220, 325–327 (1983). doi: 10.1126/science.6836275; pmid: 6836275

[114] Craciun S., Balskus E. P., Microbial conversion of choline to trimethylamine requires a glycyl radical enzyme. *Proc. Natl. Acad. Sci. U.S.A*. 109, 21307–21312 (2012). doi: 10.1073/pnas.1215689109; pmid: 23151509 PMC3535645

[115] Craciun S., Marks J. A., Balskus E. P., Characterization of choline trimethylamine-lyase expands the chemistry of glycyl radical enzymes. *ACS Chem. Biol*. 9, 1408–1413 (2014). doi: 10.1021/cb500113p; pmid: 24854437

[116] Romano K. A., Vivas E. I., Amador-Noguez D., Rey F. E., Intestinal microbiota composition modulates choline bioavailability from diet and accumulation of the proatherogenic metabolite trimethylamine-*N*-oxide. *mBio* 6, e02481-14 (2015). doi: 10.1128/mBio.02481-14; pmid: 25784704 PMC4453578

[117] Wang Z. et al., Non-lethal inhibition of gut microbial trimethylamine production for the treatment of atherosclerosis. *Cell* 163, 1585–1595 (2015). doi: 10.1016/j.cell.2015.11.055; pmid: 26687352 PMC4871610

[118] The Human Microbiome Jumpstart Reference Strains Consortium, A catalog of reference genomes from the human microbiome. *Science* 328, 994–999 (2010). doi: 10.1126/science.1183605; pmid: 20489017 PMC2940224

[119] Berry D. et al., Host-compound foraging by intestinal microbiota revealed by single-cell stable isotope probing. *Proc. Natl. Acad. Sci. U.S.A*. 110, 4720–4725 (2013). doi: 10.1073/pnas.1219247110; pmid: 23487774 PMC3607026

[120] Lasken R. S., Genomic sequencing *of* uncultured microorganisms from single cells. *Nat. Rev. Microbiol*. 10, 631–640 (2012). doi: 10.1038/nrmicro2857; pmid: 22890147

[121] Tasse L. et al., Functional metagenomics to mine the human gut microbiome for dietary fiber catabolic enzymes. *Genome Res*. 20, 1605–1612 (2010). doi: 10.1101/gr.108332.110; pmid: 20841432 PMC2963823

[122] Li M. et al., Symbiotic gut microbes modulate human metabolic phenotypes. *Proc. Natl. Acad. Sci. U.S.A*. 105, 2117–2122 (2008). doi: 10.1073/pnas.0712038105; pmid: 18252821 PMC2538887

[123] Gerlt J. A. et al., Enzyme Function Initiative-Enzyme Similarity Tool (EFI-EST): A web tool for generating protein sequence similarity networks. *Biochim. Biophys. Acta* 1854, 1019–1037 (2015). doi: 10.1016/j.bbapap.2015.04.015; pmid: 25900361 PMC4457552

[124] Kolmeder C. A. et al., Comparative metaproteomics and diversity analysis of human intestinal microbiota testifies for its temporal stability and expression of core functions. *PLOS ONE* 7, e29913 (2012). doi: 10.1371/journal.pone.0029913; pmid: 22279554 PMC3261163

[125] Zeevi D. et al., Personalized nutrition by prediction of glycemic responses. *Cell* 163, 1079–1094 (2015). doi: 10.1016/j.cell.2015.11.001; pmid: 26590418

[126] Kim K. S. et al., Associations of organochlorine pesticides and polychlorinated biphenyls in visceral vs. subcutaneous adipose tissue with type 2 diabetes and insulin resistance. *Chemosphere* 94, 151–157 (2014). doi: 10.1016/j.chemosphere.2013.09.066; pmid: 24161582

[127] Karigar C. S., Rao S. S., Role of microbial enzymes in the bioremediation of pollutants: A review. *Enzyme Res*. 2011, 805187 (2011). doi: 10.4061/2011/805187; pmid: 21912739 PMC3168789

